# Clinical trials for stem cell transplantation: when are they needed?

**DOI:** 10.1186/s13287-016-0325-0

**Published:** 2016-04-27

**Authors:** Phuc Van Pham

**Affiliations:** Laboratory of Stem Cell Research and Application, University of Science, Viet Nam National University, Ho Chi Minh City, Viet Nam

**Keywords:** Clinical trials, Stem cells, Stem cell therapy, Stem cell transplantation

## Abstract

In recent years, both stem cell research and the clinical application of these promising cells have increased rapidly. About 1000 clinical trials using stem cells have to date been performed globally. More importantly, more than 10 stem cell-based products have been approved in some countries. With the rapid growth of stem cell applications, some countries have used clinical trials as a tool to diminish the rate of clinical stem cell applications. However, the point at which stem cell clinical trials are essential remains unclear. This commentary discusses when stem cell clinical trials are essential for stem cell transplantation therapies.

## Clinical trials

Clinical trials are studies aimed at evaluating whether a proposed new therapy or drug is safe and effective. New drugs are compelled to undergo clinical trials. Clinical trials are commonly divided into four phases (phase I, II, III, and IV). However, the product is eligible for regulatory approval after just three successful phases.

## Stem cells, drugs, and stem cells as drugs

Stem cells are living cells that exist in the human body. Human growth and development originates from a single totipotent stem cell, the zygote. This stem cell proliferates continuously via two mechanisms, symmetric and asymmetric division [1, 2]. During asymmetric division in adults, stem cells ensure maintenance of important roles, in particular homeostasis. Pluripotent stem cells exist in the early stage of embryo development as the blastocyst. Almost all adult stem cells exhibit multipotent differentiation potential into a number of cell types. In the adult, the majority of tissues contain tissue-specific stem cells, with tissues rich in stem cells including bone marrow, adipose tissue, and muscle tissue, to name a few. Traditional drugs are categorized as any substance other than food used to cause a physiological change in the body. More commonly, drugs are new compounds, chemicals, or molecules that are synthesized or extracted from natural materials. However, the use of stem cells as drugs requires a new definition and approach. Stem cells as drugs or stem cell drugs are products containing live stem cells that are used as drugs.

## Stem cell therapy, personalized medicine, and stem cell drugs

Stem cell therapies are treatment procedures using stem cells. There are two types of stem cell therapy: autologous and allogeneic stem cell therapies. For autologous stem cell therapy, or so-called personalized medicine, the two subgroups include nonexpanded and expanded autologous stem cell transplantation.

Allogenic stem cell therapy includes stem cell drugs and expanded or nonexpanded allogenic stem cell therapies. The main difference between stem cell drugs and other approaches is that stem cell drugs are used to treat a large population of patients using the same source of stem cells.

## When should stem cell clinical trials not be performed?

As already explained, there are many different approaches for stem cell therapies. The objectives of clinical trials are drug safety and effectiveness evaluation. In personalized medicine, the stem cells are obtained from the patients themselves; therefore the risk of rejection is negligible. This makes the stem cells safe and nontoxic. Nonexpanded autologous stem cell therapy is the only process whereby stem cells are moved from one tissue to another within the same patient. The key difference between this approach and the use of new drugs, which are unfamiliar to the patient’s body, is therefore obvious. Consequently, the main outcome in a clinical trial for nonexpanded autologous stem cell transplantation is to investigate treatment efficacy rather than safety of the stem cells.

A key question is, if a procedure using nonexpanded autologous stem cell transplantation is successful in one country, whether it is essential to repeat the clinical trial in countries aside from the original country? In my opinion, repeating the clinical trial across different countries should not be requisite. At present, there are no findings that can be recorded from these clinical trials.

## Why we should not have to repeat clinical trials

Autologous stem cells are very safe. To date, there are no publications which present stem cells as harmful. In fact, in a systematic review by Benoit et al. [3], autologous cell therapy in critical limb ischemia was found to be 100 % safe in treated patients. Meta-analysis of autologous stem cell transplantation for the treatment of limb ischemia similarly showed a 100 % safety success rate [4]. Meta-analysis of autologous stem cell transplantation for the treatment of patients with type 1 and type 2 diabetes mellitus has also shown a 100 % safety rate [5, 6]. Furthermore, autologous stem cell therapy for end-stage liver cirrhosis [7], osteoarthritis [8], and limbal stem cell deficiency [9] is reported to be 100 % safe. Subsequent clinical trials using autologous stem cell transplantation will therefore also determine that this cell source is 100 % safe.

With respect to autologous patients, some countries usually apply to repeat clinical trials for new drugs and for imported approved drugs. Indeed, they need to check the pharmacology of the drugs within the local patient population. However, for autologous stem cell therapy, the “drug” is from the patients themselves. Although the quality of the stem cell drug may differ between patients, the pharmacological effects may also differ in different patients. We therefore cannot control the quality of autologous stem cell products, meaning that efficacy may also differ between patients.

## When should we perform clinical trials?

Stem cell clinical trials should be carried out in the following highlighted cases:

1. A clinical trial can be essential for stem cell drugs. The quality of the stem cell drug must be maintained, controlled, and stable. The stem cell drug will be used in many different patients (a population); with each patient, the eliciting response differs using the same drug. The clinical trial should therefore be carried out to identify new findings for safety or efficacy in a certain population. Similarly, some clinical trials also can be repeated if there is any modification that can directly affect stem cell quality.
2. Clinical trials may be essential when using allogenic stem cell transplantation. However, stem cell quality can differ between donors. The findings from one donor patient cannot therefore always be applied to the recipient patient population.
3. Clinical trials should be carried out using both autologous and allogeneic expanded stem cell therapies. However, we need to understand that the main objective of the clinical trial is for evaluation of the culture protocol and not the stem cells; for example, whether ex-vivo culture protocols affect the stem cell genome stability*.*

## Conclusions

Clinical trials are performed to test the safety and efficacy for a proposed new therapy or drug. However, because they are different from the traditional definition of a drug, in some cases stem cells are drugs but in other cases they represent treatment processes similar to autologous skin grafts. It is therefore nonessential to repeat clinical trials for nonexpanded autologous stem cell therapies. In fact, there are no new findings regarding autologous stem cell safety or efficacy following clinical trial repeats. Clinical trials should be carried out only in some stem cell drugs, or in cases that could cause changes in the phenotype or genotype of the stem cells.
